# Additional Notes on Provincial Asylums for the Insane in France; with a Brief Report of the Institution at Illnau, in the Grand Duchy of Baden

**Published:** 1852-07-01

**Authors:** John Webster

**Affiliations:** FELLOW OF THE ROYAL COLLEGE OF PHYSICIANS; CONSULTING PHYSICIAN TO ST. GEORGE AND ST. JAMES'S DISPENSARY, ETC.


### Original Communications.

### ADDITIONAL NOTES ON PROVINCIAL ASYLUMS FOR THE INSANE IN FRANCE; WITH A BRIEF REPORT OF THE INSTITUTION AT ILLNAU, IN THE GRAND DUCHY OF BADEN.

BY JOHN WEBSTER, M.D., F.R.S., FELLOW OF THE ROYAL COLLEGE OF PHYSICIANS; CONSULTING PHYSICIAN TO ST. GEORGE AND ST. JAMES'S DISPENSARY, ETC.

(*Concluded from page* 255.)

### STÉPHANSFELD ASYLUM.

THIS public institution—appropriated for the reception of indigent lunatics belonging to the two departments of the Upper and Lower Rhine—is situated on a sandy, well-aired, and open plain, near the commune, or small town of Brumath, lying about ten miles north-east of Strasbourg, and adjoining the railway leading from thence to Nancy by Sarrebourg. Previous to 1836, departmental insane patients were usually consigned to the city general hospital, or Hotel Dieu, where they lived in a most deplorable condition, having neither garden nor court-yard for recreation; and, although some of these unfortunate inmates were most improperly allowed to associate with ordinary hospital patients, a large proportion always remained in rigid confinement. Since the period when Esquirol pointed out, and condemned these improper proceedings, the new asylum of Stéphansfeld has been constructed; which is truly, and without exaggeration, highly creditable to the public authorities of Alsace. The situation selected occupies a level country, which affords varied but agreeable prospects on several sides, with a forest, at a little distance, fronting the façade and main entrance. Being thus pleasantly situated, and not overlooked by neighbours, the asylum exhibits altogether an agreeable appearance. In addition to these advantages, as the space occupied by the dormitories and court-yards is ample, whilst the latter are airy and spacious, visitors very soon become favourably impressed with the superior capabilities this establishment possesses, particularly, when compared with various more anciently constructed institutions.

Unlike many public asylums in England, instead of being one large building, having occasionally a palatial elevation—which attribute, however pleasing it may appear to spectators from without, does not augment, nay, even sometimes diminishes the residents' comfort, who are thus sacrificed, as it were, to architectural display and ornament—Stéphansfeld consists of a large central structure, containing the chapel and official residences; from whence various dormitories, having their respective court-yards, branch off on each side. The division for male patients lies on the right hand: whilst that for females occupies the opposite. The sitting or work rooms are large, lofty, exceedingly well ventilated, and conveniently arranged; and the dormitories being equally spacious and well aired, with windows on each side, the entire arrangements really looked cheerful. No iron bars appeared anywhere; and, as every room possessed an agreeable, if not always an extensive view of adjacent gardens or fields, few structures could be better adapted for the objects proposed than the new sleeping-rooms and court-yards which had been recently completed. The place really looked more like a factory, or rather an extensive agricultural establishment, than a madhouse. Additions, with varied improvements, were still in progress; and, amongst the former, it may be stated that a new building intended to contain numerous workshops for employing patients had been commenced only very recently, whilst the dairy-house, and an inclosure for feeding pigs, would be soon enlarged. Additional gardens were likewise projected, and a mount for patients was being constructed in an adjacent field. In short, activity and judicious advancement in the right direction seemed everywhere observable.

Each court-yard being large, and ornamented by parterres of flowers or trees, formed agreeable promenades for patients; and, as the adjoining fields were visible through open railings, whilst the sunk fence, or intervening hedges, did not obstruct the prospect thereby afforded, inmates could see several miles into the country: or, if they ascended any mount made in nearly every inclosure. the high Schwarzwald mountains of Baden, beyond the Rhine, might be distinguished. Even in the division appropriated for excited lunatics, shrubs, trees, and flowers, embellished the court-yards: where, it should be mentioned, very little damage is seldom if ever committed, particularly on the male side. The cells for secluding dangerous or agitated patients appeared few in number, but even these apartments rarely had occupants. Their construction was, however, so very superior to any heretofore inspected that some special notice thereof becomes appropriate, along with a strong recommendation to construct similar rooms in other establishments. Each cell was cheerful, well ventilated, and had two opposite doors. Besides the above, they also possessed separate skylights, which opened into a large upper apartment, only used as a store, and where one attendant could easily overlook the entire series of apartments, should all be ever in requisition at the same time. These openings were protected by an open wire frame, and had also a sash-window containing variously coloured glass: and both being moveable, either could be used as seemed most expedient, whilst any interference by patients was impossible. The soothing effect produced by variegated light shining through such skylights into the space below, made it appear more like a boudoir for repose, than a cell for the temporary seclusion of excited maniacs. Indeed, the arrangement appeared novel, whilst its application seemed judicious.

When I visited Stéphansfeld last September, the total resident lunatics amounted to 441; comprising 220 male, and 221 female patients. Amongst the above number, 61 were classed as pensioners—34 males and 27 females—who paid from 400 to 2400 francs annually; with 657 francs additional, when the inmate had also an attendant. Besides these 441 lunatics, if the officers and every other person resident within the premises be enumerated, the entire population would then comprise 517 individuals. Believing it may prove interesting to give some details respecting the 76 officials constituting the executive of this asylum, in order to describe, as it were, the effective machinery moving so large an establishment, I would briefly remark that, 18 were sisters of charity, belonging to the religious order "St. Vincent de Paule;" 35 were male and female attendants, including 4 superintendents; whilst 17 persons acted as gardeners, labourers, barbers, dairymen, or in other menial capacities. To these must be added the director, 3 medical attendants, the receiver, and a steward, making in all 6 officers who superintended their respective departments.

The two districts to which the indigent lunatics of this asylum chiefly belong, viz., the Upper and Lower Rhine, being different in various respects from other departments of France, I am therefore led to suppose, a few particulars respecting several existing characteristics may be interesting. For instance, in this province, which constituted ancient Alsace, the German and French languages, are generally spoken; its population comprise both Catholics and Protestants, whilst many of the inhabitants may be denominated a mixed race, seeing they are often the descendants of French and German progenitors.

Nevertheless, considering it would be rather out of place to discuss, at any length, how the influential causes here shadowed forth may have affected the general population, I will merely allude, at present, to one or two interesting peculiarities noticed amongst the insane residents of Stéphansfeld; in order to illustrate several features, whereby other foreigners may be able, perhaps, to unravel questions which otherwise seem intricate, if not obscure.

My present object being rather to record facts than to enunciate speculative opinions, or even to deduce general inferences, unsupported by sufficient evidence, I at once proceed to state that, amongst the whole insane residents under treatment, at the period this asylum was inspected, 308 were Catholics, comprising 151 male and 157 female patients; 111 belonged to the Protestant church, 54 being males and 57 females; 21 were of the Jewish persuasion, consisting of 14 male and 7 female patients; and lastly, 1 male was an Anabaptist. With reference to the language of residents, 224 persons, both sexes included, spoke German exclusively; 47 could only speak French; and 163 were able to converse in both these dialects indiscriminately; the other 7 inmates being idiots or dumb persons. Again, in regard to the elementary parts of education, 201 were able to read German, 38 only French, and 131 read both languages; which left 71 individuals utterly unable to decipher any book whatever. Lastly, respecting caligraphy, 168 wrote German, 34 French, and 125 could do so in either language; whilst the remaining 114 were wholly ignorant of that useful qualification. Respecting the social position of the lunatics under treatment, it is interesting to remark that, 295 were single, comprising 156 male and 139 female patients; about one fourth, or 105 were married persons, including 55 men and 50 women; besides which there were 9 widowers, and 32 widows. Again, as to age, it deserves notice that, 23 male and 26 female inmates were less than 30 years old; 153 men and 121 women, or upwards of 62 per cent. of the entire insane population, ranged from 30 to 50 years of age; 40 men and 66 women were between 50 and 70; whilst 8 female and only four male lunatics had passed the latter period. According to these authentic data, it therefore appears that, insanity attacks the male part of the population in Alsace more frequently during the prime of life, than it affects females; whereas, when young, or arrived at advanced years, the proportion of women labouring under mental maladies is comparatively larger than of the other sex, and they also live longer, although insane.

Occupying the lunatics being a prominent feature in the system pursued at the Stéphansfeld institution, it cannot but prove highly interesting to all advocates of similar proceedings, to learn some details respecting the employments usually patronised. To illustrate this important point, I select the day previous to my visit, when the official report of the different occupations contained the following statement. The total number of patients then employed amounted actually to one half, or 220 individuals, who were thus distributed:—Forty-seven women were engaged in sewing or mending clothes, 27 in knitting, 16 in the culinary department, 14 in domestic duties, 12 in the laundry, 4 in spinning, and 2 in making list-slippers; thereby giving 122 females at work, out of the 221 inmates of that sex. Amongst the 220 male lunatics 98 were occupied; of whom 24 acted as servants in different divisions, 18 worked in the garden, 16 were cutting and storing firewood, 8 were engaged in digging the foundation of new workshops, 7 in cleaning the premises, 6 laboured at the farm, 4 in the stable, 4 in carding wool and hair for mattrasses, 3 worked as masons, 2 assisted in the kitchen, 2 were busy at bookbinding, 2 in weaving, 1 was mending clothes, and 1 acted as bath-house attendant. In addition to the 98 lunatics employed as now detailed, it ought to be further mentioned that, 8 male patients were then enjoying a promenade by way of recreation in the adjoining forest. Perhaps the above minutiæ may appear tiresome, although instructive; but believing they will convey some notion of the manner in which the labour system is carried forward in a large French lunatic asylum, I have been induced to state various details, even although doing so may be considered as somewhat supererogatory. To most minds, few spectacles seems more interesting than to contemplate frail human beings deprived of reason, and separated from the active world without, thus busily and often agreeably engaged; seeing their attention is thereby occupied, whilst listless time does not then hang so heavily on their hands. Besides which advantages, physical health being always promoted, the patient's mental malady often derives decided amelioration.

Having a farm of 62 acres belonging to the asylum, this, with extensive gardens adjoining, supply ample scope for out-door occupations. After considerable experience, the director, M. Richards, and Dr. Dagonet, the physician, are both strong advocates for employing lunatics in the open air, especially by means of agricultural and horticultural labour. The latter officer considers such employments often produce real benefit to insane patients; since in some, out-door work essentially promotes recovery, whilst in many, physical exertion materially improves their bodily health. Seeing a large proportion of the lunatic inmates—about four-fifths—are incurable, it consequently becomes a matter of essential importance how to improve their bodily condition; hence order, labour, and discipline, constitute essential adjuvants; at the same time that judicious hygienic measures are also put in requisition.

As might be expected, after perusing my previous remarks, readers will likely anticipate that most inmates of this asylum generally enjoyed good bodily health. Such was the fact: and very few patients occupied the infirmary. One peculiar adjunct to the female sick ward here deserves special notice, from its novelty and really useful purposes;—namely, an airy and spacious gallery or verandah, which could be shut up close, or freely exposed to the external atmosphere, as circumstances might require. Being on the same level with the infirmary, patients who were improving in health, and for whose complaint a change from the confined air of their ordinary dormitory to an open atmosphere, with moderate bodily exercise, would be advisable, might here obtain both, without descending stairs, or incurring the fatigue of making much alteration in their exterior appearance or habiliments. The important benefits derived from such a succursal apartment, for persons approaching convalescence, are so evident that, the same plan might be very judiciously adopted at other institutions for the insane, as also in hospitals and infirmaries.*

Respecting the application of personal restraint in an asylum where so much liberty is otherwise enjoyed by patients, the cheerful appearance of the entire

* Being much impressed with the many advantages convalescent patients would derive from having an apartment like the one alluded to, and in which they could at any time, when considered advisable, take bodily exercise, or inhale an atmosphere entirely different from the confined and often vitiated air of their ordinary dormitories, I mentioned to Mr. Johnson, the treasurer, and committee of Bethlem Hospital, the above novel feature characterising Stéphansfeld, at the same time recommending its adoption as an appendage to the new infirmaries about to be erected. My proposition seemed so satisfactory, that Mr. Smirke, the able hospital architect, was requested to prepare plans accordingly. These were in consequence made, and subsequently approved; whilst contracts have been since accepted for erecting suitable infirmaries, including two promenade apartments or verandahs. The contemplated expenditure will amount to about 5,000*l*. But the governors being always anxious to ameliorate in every way the afflictions, whether physical or mental, of inmates sent to this institution, expense is never considered, if necessary or judicious. Desirous of conveying to readers of these notes some general idea of the proposed erection, I wrote to Mr. Smirke requesting he would kindly favour me with a establishment, and the principles actuating its executive, must have already led to the surmise that, any kind of physical coercion, or the use of camisoles are very rarely employed. Such was the case; and it is highly satisfactory to report, on the day of my visit, no female patient was in any manner restrained, throughout the entire establishment. One man was, however, partially confined by a camisole; but in this case it ought to be added that, the restraint used was chiefly employed to prevent the patient from deranging the dressings applied to one of his legs recently broken. Notwithstanding this solitary example of partial bodily coercion, Stéphansfeld asylum stands prominently forward as a gratifying illustration, not only in reference to the decided disuse of physical restraint, but it may likewise be quoted as remarkable for the tranquillity very generally prevalent throughout the institution; particularly, amongst insane female patients, who certainly are often, especially in the southern and central departments of France, much more noisy and excitable than in either Alsace or Lorraine.

The medical staff of this institution consists of one physician, Dr. Dagonet, who is a practitioner of much promise, and well known to the profession. He was very recently an interne at Maréville, and obtained his present appointment through merit. There are besides two internes, one of whom is likewise a Doctor of Medicine. This gentleman, Dr. Weill, had performed his own duties so *well* and assiduously that, he was charged with the whole medical superintendence of the establishment, during Dr. Dagonet's recent absence at London, where he had gone to visit the great Exhibition.

For 1850, the following statistical return exhibits the movement of patients at the Stéphansfeld asylum:—

| | Males. | Females. | Total. |
|---|---|---|---|
| Admitted | 82 | 73 | 155 |
| Discharged cured | 17 | 18 | 35 |
| Died | 17 | 15 | 32 |

Amongst the 155 lunatics admitted, 100 belonged to the Lower, but only 43 to the Upper Rhine department; the remaining 12 patients being from other districts. This fact either shows the greater prevalence of insanity in the former department, or that the public authorities were more assiduous in sending recent cases to the asylum, contrasted with those of the Upper Rhine; from whence a large proportion of the insane persons received were chronic brief outline of the building it was intended to construct. The subjoined is that gentleman's reply:

"Berkeley-square, May 18, 1852.

"Dear Dr. Webster,—The following short description will explain the nature of the provision soon to be made at Bethlem Hospital, for enabling convalescent patients to take bodily exercise in the open air, before they can be allowed to go out of doors. On the upper story of the infirmary about to be built, on both the male and female sides, there will be an apartment 41 feet by 24, and 13 feet high, reached by stairs leading from the rooms appropriated as infirmaries. The roof is of copper, with a plaster ceiling: three sides of the apartment are wholly of glass, with iron pillars and sashes. The glazing consists of long narrow vertical louvres of glass, each of which turns on a centre, so that they can be all opened, wholly or in part,—one movement opening sixteen louvres simultaneously—much in the manner of common parlour blinds: the clear space between each louvre, when open, is about five inches: by this arrangement all three sides of the apartment will be perfectly open for the admission of fresh air, without any possibility of danger to the patients. The bottom of these glazed louvres is three feet from the floor, and they extend up to the ceiling. Rain cannot enter, and the sunshine may be modified, if necessary.

Very faithfully yours,

"Dr. Webster, &c." "SYDNEY SMIRKE."

cases, of whom nearly all exhibited very little prospect of ultimate recovery. Respecting the varieties of mental disease they manifested, 61 of the cases admitted—or two-fifths of the entire number—were attacked by mania, 37 laboured under dementia, 37 suffered from lypemania, 8 were monomaniacs, 8 epileptic patients, one was an idiot, whilst the disease of the remaining three inmates had no specific denomination. According to these official data, it appears evident a large proportion of the residents were incurable patients; especially, seeing one third of the whole number suffered from dementia, or epilepsy, few or none of whom afforded the slightest prospect of recovery, or even of any improvement.

Adding the 372 insane patients remaining under treatment, on the 1st of January, 1850, to the 155 admitted during that year, the following figures indicate the various types of mental maladies by which the 527 cases thus enumerated were afflicted. It should likewise be remembered, as unfortunately every year, most of the new admissions appeared of a very hopeless description, from including idiots, epileptics, and demented persons, about two-thirds of those enumerated in the table were considered incurable.

*Type of Mental Disease affecting* 527 *Lunatics under Treatment during* 1850 *at the Stéphansfeld Asylum :*

| DISEASE. | M. | F. | TOTAL. |
|---|---|---|---|
| Mania . . . . . . . . . . . | 55 | 101 | 156 |
| Lypemania . . . . . . . . . | 33 | 43 | 76 |
| Monomania . . . . . . . . . | 25 | 17 | 42 |
| Dementia . . . . . . . . . . . | 95 | 85 | 180 |
| Mania, complicated with Epilepsy . . | 30 | 9 | 39 |
| Imbecility and Idiocy . . . . . . | 15 | 19 | 34 |
| Total under Treatment . . . | 253 | 274 | 527 |

According to the above statement—which I compiled from official documents —it appears mania and lypemania were more common in female than male patients; whereas dementia, monomania, and mania, complicated with epilepsy, oftener affected men than women; whilst imbecility and idiocy ranged nearly equal in both sexes. Viewed in the aggregate, female patients predominated over male inmates; the excess of the former being 21, or 8·30 per hundred cases then under treatment. This fact deserves remark, as insanity is believed to prevail more frequently amongst males than females in the northern departments of France; which opinion is, however, not supported invariably by the evidence I have been yet enabled to compile from various sources. The large number of epileptic patients, in the male division, likewise merits special notice; more particularly, when contrasted with the much smaller ratio of similar cases amongst female inmates. Thus, 1 person in nearly every eight male patients was affected by epilepsy, contradistinguished to one case of the same severe disease recorded in about 30 female lunatics. Consequently, that malady proved nearly four times more frequent amongst the former, than the latter sex.

All practitioners conversant with mental diseases know, from experience, that the sooner an insane patient is placed under judicious medical and moral treatment, the greater probability prevails of subsequent recovery. This axiom being well established, and universally admitted, scarcely requires any proof or evidence. Nevertheless, it may be now stated, in corroboration of the above opinion that, the results obtained at Stéphansfeld fully bear out such conclusion. For example, of the 35 patients discharged cured during 1850, more than half, or 20, left the institution convalescent before their malady had continued three months. Age likewise materially influenced similar favourable results; seeing 4 were cured before they were 20 years old; 8 from 20 to 30; 21, or two-thirds, ranged from the latter period to 60; whilst only two persons seemed restored to sound mental health, who had completed their fiftieth year. Based upon these statements, a physician may therefore safely infer, the older a patient has become, and the longer an attack of insanity may have continued, so much more likely should ultimate recovery be reckoned improbable.

In referring to the various forms of mental disease affecting the 32 patients whose deaths were recorded, it appeared that, 15 laboured under dementia, 10 died from mania, 4 by lypemania, 2 from monomania, and 1 by epilepsy. Again, respecting the duration of their treatment in the asylum; one-third of the cases, or 12 patients, died under a residence of three months, 6 from that period to six months, whilst in the remaining 14 deaths, the party had resided at least one year. Another point of importance also deserves special notice, since it materially concerns the medical treatment of insane patients; namely, the nature of the bodily disease from which death evidently ensued, in the whole 32 fatal cases now recorded. On this instructive subject, the register of autopsies kept at the asylum supplies ample information, and from which I quote the following details:

According to that authentic record, 11 patients died through affections of the head and nervous system; of whom 5 were reported in consequence of apoplexy, 3 from softening, and 3 through inflammation of the brain. Again, 12 inmates died from pectoral disease; amongst whom 7 deaths arose from consumption, 4 by inflammation, and 1 through gangrene of the lungs; whilst 7 cases terminated fatally from disease of the abdominal viscera. Besides these deaths, two fatal casualties are classed under the denomination of "asphyxia by suffocation."

Unlike the results recorded at several asylums, especially that of Dijon, which I especially mentioned in a previous page, the above details show the great frequency of phthisis as one of the diseases proving very fatal amongst the Stéphansfeld patients, seeing 7 cases were reported by that malady, whereby it occupied the highest position in the mortuary scale. In every example of that description, considerable disorganization of the lungs was observed, whilst the paranchyma exhibited large purulent cavities, exhaling a fœtid odour. Regarding the pathology of insanity considered as a general question, and in order to aid other investigators, who have most laudably endeavoured to associate the different morbid appearances observed on post mortem examinations, with the symptoms previously observed, and thus foretel from the specific type of mental affection manifested during the patient's life time, those diseased structures which would be found on dissection, the following valuable reports illustrating so very difficult a subject, lately made by Dr. Dagonet in reference to the pathological examinations recorded at the Stéphansfeld asylum, deserve mention. From the autopsies performed in 1850, tubercles appeared in five instances of mania, in one of monomania, and in another case of dementia; whereas, pneumonia seemed to occur indiscriminately. On the other hand, softening of the brain always supervened in patients affected with mania, dementia, or epilepsy; whilst, in three cases of fatal meningitis, two laboured under mania, and the third had dementia.

Formerly, intermittent fever and intestinal affections occurred more frequently in the asylum and its neighbourhood, than of late years. This result arose, in great part, through much new ground having been exposed to solar action conjoined with moisture, during the construction of the Paris railway; but especially, from excavating the canal betwixt the Marne and Rhine. The consequences thus produced were particularly disastrous to the inmates of Stéphansfeld during 1847, as also in several other districts of Alsace: which were literally ravaged by these diseases, almost like a pestilence. For instance, in the commune of Bollwiller—situated in the Upper Rhine, and having 1400 inhabitants—not less than 1103 persons were attacked by ague. Although the railroad and canal are now both completed, still, the latter not being yet opened for traffic, and as it contains several stagnant pools from whence malaria is said to emanate, considerable predisposition to intermittent fever prevailed last year; but only then amongst patients previously attacked by that disease, all other persons having remained unaffected.

Considering some account of the causes which apparently produced attacks of insanity in the 155 cases admitted last year, at this institution, may be instructive, it should be stated that, 26 patients became insane through grief or anxiety, 8 from the passion of love, and 8 by religious fears: thus making 42 cases of madness produced by moral influences. From physical causes, the number of cases amounted to 43, of which 21 were in consequence of bodily disease, 17 arose from intoxication, and 5 through sensual excess. Again, from hereditary tendency, 35 cases were reported: thereby leaving 35 patients in whom the apparent cause was not correctly ascertained. One point in these statistical details, however, deserves special remark, namely,—the number of instances where drunkenness is stated to have produced attacks of mania. To find so many as 17 persons, out of 155 admissions, lose their reason by intoxicating spirituous liquors, certainly furnishes strongly condemnatory evidence respecting the intemperate and irregular habits of the lower classes resident in this province. That 8 individuals became actually insane through religious fears is, however, not surprising, considering the different sects resident in this part of France; seeing controversies on sacred subjects are by no means uncommon, where Catholics and Protestants are thus often placed inimically in juxtaposition, and whilst they frequently entertain very opposite sentiments respecting questions of the greatest import to man's present welfare, and future salvation.

According to Dr. Dagonet, religious melancholy seems to have become more frequent at Stéphansfeld than at any other French asylum for the insane. This unfortunate result, no doubt, arises from the superstitious practices and erroneous notions often prevalent amongst the ignorant portion of the Alsacian population. In proof of such remarks, I would refer to a recent official report, because it expresses the opinion of a physician who is fully competent to speak on these subjects, both from ample personal experience and intimate knowledge of his countrymen. Dr. Dagonet says, in reference to this matter, "The numerous religious sects domiciled face to face in the two departments of Alsace, and who are thereby constantly in communication with each other, occasionally engender troubles and disorder in certain localities. Passion produces a state of irritation which, by repetition, goes so far in some cases as to affect reason: especially, as it is known that religious divisions, even more than political dissensions, awaken the most violent feelings, where superstition has taken a strong hold upon the susceptible minds of a large number of the rural population." No observations could be more explicit or decisive; and, coming from such an authority, any further argument respecting similar questions appears superfluous.

Hereditary tendency to insanity seems likewise to have materially promoted the accession of madness in numerous cases admitted, which influence always acts more powerfully upon the human frame, when conjoined with other exciting causes. Under this category, two very melancholy illustrations of hereditary predisposition to mania, affecting particular families, occurred some time ago at this asylum, which deserve record, on account of the number of persons who became attacked. In one of the families, originally consisting of seven children, it happened that two members came, during the same time, under treatment at Stéphansfeld, after three other relatives of the identical stock had died insane. In the second example referred to at present, two twins were also inmates along with the previous patient, so that both instances appeared more remarkable from appearing, as it were, consentaneously. Taking the above facts into consideration, besides many similar examples met with in almost every lunatic asylum, it cannot be too strongly urged upon all parties, whether private individuals or legislators, the absolute necessity, nay, imperative duty, of always discouraging the intermarriage of members belonging to any family, in which decided hereditary tendency to insanity prevails. Cases of that description, where one side of the house is tainted seem bad enough, although such calamities may be often greatly ameliorated by proper education, as also through judicious management; but if two parents, equally affected with hereditary predisposition to madness have offspring, the ulterior consequences frequently become most calamitous. Indeed, so many serious social evils may thus supervene that, it would be humane towards individuals, and certainly more beneficial to the community, were these unions always interdicted. In Great Britain, the legislature have, very properly, enacted and said, lunatics shall not dispose of any property by will, nor be allowed to execute legal documents, and cannot be punished for crimes, even of the greatest enormity; how much more necessary, then, does it not appear for efficient steps being taken to prevent the occurrence of so great a calamity as the former contingency!

In order still further to exemplify the disastrous consequences often supervening through hereditary tendency to mania, it should be added that, during last year, there were under treatment at Stéphansfeld—1. a mother and daughter; 2, a brother and sister; 3, two sisters; 4, three sisters; 5, two cousins; 6, an aunt and niece: 7, a religious monomoniac female, in whose family seven relatives were actually insane; and, 8, a husband and wife, who, although not relations by blood, in regard to their descendants were perhaps even worse. The melancholy illustration previously quoted, of three sisters being under medical treatment, appears so very remarkable a coincidence as to deserve special notice, particularly as they all arrived at the asylum on the same day, and had been seized with mental alienation almost simultaneously. The above patients were members of a numerous family who exhibited a strongly marked hereditary predisposition to lunacy, and had been, it was reported, unfortunately led astray by deep but mistaken devotional feelings: or rather, to speak more correctly, through their excessive superstition. Amongst other fancies, they held frequent conferences with wandering gipsies who pretended to prognosticate future events, and whose confident predictions—however absurd, they implicitly believed; consequently, the credulous dispositions of these poor creatures were taken advantage of by cheating jugglers and mountebanks, even now often met with in many rural districts of Alsace, where they practise their tricks and avocations upon the ignorant populace. Having become the victims of strange delusions, the sisters began to pray together, and to perform various mystical ceremonies, whereby their fanatical exaltations augmented in force more and more every day, until one of them actually believed she was the Virgin Mary, and hence insisted upon the other two acknowledging the accuracy of her conviction. Subsequently, the youngest sister being supposed enchanted, or possessed by some demon, she, in consequence, became the object of such excessive personal violence and outrage, on the part of her two relatives, that death followed very soon afterwards. In these sad cases, now related, hereditary tendency to madness, and superstitious ideas were materially influenced by that predisposition to irritation, which so often exercises considerable power over individuals, as even to produce something like contagion, more especially in excitable temperaments, or delicate physical organizations; whereof, marked and instructive examples are occasionally recorded in the annals of science.

The above deplorable history, and its concomitant evil consequences—derived from an authentic document—has been thus minutely reported, in order to exemplify the baneful results sometimes following fanatical notions, when acting upon credulous imaginations; especially, where the parties implicated unfortunately had decided hereditary predisposition to mental disease. Besides being highly instructive, on account of that peculiar feature, and the violent symptoms each of the three cases portrayed, they also furnish most important evidence with reference to the difficult legal question adverted to in a previous paragraph.

When perambulating the different apartments of this asylum, I was much pleased on remarking the elegance with which several dormitories and day-rooms were embellished. Instead of showing dead unmeaning walls, which elsewhere possessed no attraction for the eyes of even a passing stranger, and much less residents, various apartments were tastefully covered with ornamental paper, which produced, through the objects there delineated, often pleasing impressions upon the minds of spectators. This agreeable feature may be best exemplified by briefly describing one of the female work or sitting rooms which I visited. In this apartment, each of the four walls exhibited views of beautiful country scenery. One was a landscape of some place in France, another contained a view of Switzerland, and a third represented romantic looking lakes and a valley in Scotland, where stalwart highlanders—wearing bonnets and dressed in tartan philabegs—seemed gaily sporting over their well known land of the mountain and the flood. At one side of this agreeable room, an elegant clock not only indicated the hour to the different inmates then busy at work, but it played an exhilarating tune on our entrance, which could be at any time varied, or repeated, by touching a spring to set the machinery again in motion. Other ornaments I noticed might be also mentioned, but it seems unnecessary: however, one feature of a temporary character should not be forgotten on this occasion, as the circumstance was exceedingly pleasing to witness, besides indicating much good feeling which seemed to actuate the inmates. In the centre of this apartment, a kind of drawing, or picture, had been made with sand of different colours, but so arranged as to look like wreaths of flowers, in the same manner as London ball-rooms are chalked when a gay party assembles. In the middle of these arrangements, which had been all made by inmates, the words, "Vive la famille Dagonet," were accurately traced in variously coloured sand. This motto was intended by the female lunatics occupying that division as a welcome to their worthy physician, who had only returned the previous night from visiting the British metropolis; and, I must add, the compliment thus paid was highly creditable to all parties concerned.

Throughout the entire establishment, great cleanliness and apparent comfort prevailed. Ventilation was excellent; whilst the inmates everywhere conducted themselves with order and quietude, all having an appearance of being contented, quite as much as lunatics could be expected to exhibit in their peculiar position. The bedsteads were generally made of wood, but iron were also used, especially for dirty patients. None of the dormitories appeared crowded, although some will be considerably relieved in that respect, by transferring a section of the private patients to their new residence, so soon as the building now in progress shall be completed. When this is done, a separate court-yard will be also appropriated for the epileptic patients, who are at present rather numerous.

Exercise in the open air being considered most essential, and, as it often proves highly beneficial when treating insane patients, the farm now belonging to the institution affords an excellent locality for carrying out that principle.

Besides these means of employing patients in out-door labour, the gardens, piggery, and cow-house—immediately adjoining the asylum, greatly conduce towards attaining the same object. According to the extended experience, both of the physician and director, bodily labour in the open air is found to be, in many instances, of real benefit to the insane. In some cases, it becomes an important means of cure; in others, the exercise improves their physical health; and, in many, it even appears to be a fruitful source of contentment. Such gratifying effects are frequently noticed at this asylum, where a number of inmates may be seen engaged in different kinds of work; and often with as much, if not even sometimes more assiduity than ordinary workpeople.

Physical labour is, however, not the exclusive kind of employment encouraged at this asylum, and patronized with zealous energy. Intellectual exercises of various kinds being, likewise frequently and systematically, brought into operation; for which purpose, an able schoolmaster has been especially appointed on the male side, whilst one of the sisters of charity acts as the teacher in the female division. Through the assiduous exertions of both these efficient and most useful assistants—who always act under the superintendence of the physician—different kinds of intellectual exercises are daily put in requisition; which occupy the lunatic's attention, and thereby withdraw the patient's weakened mind, if possible, from contemplating those morbid ideas, or delusive fancies, which characterize, or appertain to the particular form of mental malady then present. Having that object constantly in view during the treatment, conversations on history, instruction respecting the physical and natural sciences, translating interesting works, analyzing instructive publications, reading aloud, and, lastly, exercising the memory by repeating from authors passages previously learned, are zealously promoted as the chief means likely to fix the mobile mind of an insane person. Other patients, less advanced, are first taught the elementary parts of education, in a somewhat similar method to that already mentioned as now adopted at Armentières and Auxerre, with so much advantage.

Besides these often useful adjuvants in the management of lunatics, music and singing have also been frequently employed with very beneficial consequences, whilst even plays were acted on two occasions, one by female, the other by male lunatics, who appeared as performers. Respecting the latter kind of amusement, I can say nothing from individual experience, never having been present at any of these histrionic entertainments; nevertheless, their utility seems doubtful, if the effect be not injurious. In reference, however, to the former, and correctly speaking, certainly more intellectual occupations, I can assert with some confidence, after personally witnessing both male and female insane residents, in their respective school-rooms, when occupied with the tasks assigned, that the impression made upon the audience then present appeared beneficial, and must have produced sanative consequences. In one of the apartments I visited, whilst these exercises were proceeding, about forty insane pupils had assembled. Some read aloud, whilst others listened; several afterwards recited; then a party sang in chorus, accompanied by the teacher on a fiddle; and, lastly, questions in arithmetic were asked, to which, if one lunatic could not answer correctly, another was requested to reply; two inmates were also making drawings at a separate table, and I would add, that one of the monitors, who appeared at the time particularly zealous in teaching several pupils placed under his special superintendence, was himself a lunatic. Altogether, the scene here exhibited appeared most interesting, and was also exceedingly creditable to the asylum executive authorities. In carrying forward the varied intellectual occupations, only now briefly described, the director and physician—who both take the liveliest interest in the scheme pursued and its success—are most ably assisted in their endeavours to improve the condition of numerous lunatic inmates under treatment, by the meritorious exertions of M. Grucker, the present schoolmaster, who is deservedly esteemed as one of the most efficient officers in this establishment.

After passing nearly an entire day, greatly to my own satisfaction and improvement, in the company of M. Richard and Dr. Dagonet, whereby I gained much practical and valuable information respecting the excellent asylum under their respective management, I left the above-named gentlemen late in the afternoon, and returned by railway to Strasbourg.

However, before concluding the report of my visit to Stéphansfeld and its active executive, of whose courtesy, kindness, and civility, the most agreeable reminiscences will always continue; one important, although final remark, must be made, namely,—whatever sentiments may have been excited in Esquirol's mind on inspecting the former and very objectionable receptacle for lunatics, in the ancient capital of Alsace, these feelings would be no longer applicable. Were that eminent physician and philanthropist now alive, or could inspect the new asylum in this district of France, assuredly, any anathema formerly expressed by so very high an authority, would be amply neutralized by the decided approval which he, doubtless, would then proclaim respecting various internal capabilities characterizing the present building: as also in reference to many benevolent exertions recently and successfully made in order to improve the mental condition, besides materially to augment the bodily comforts and social condition of its often psychically afflicted insane population.

### ILLNAU ASYLUM.

Having arrived near the confines of Germany, and knowing the institution for lunatics recently established at Illnau, in the Grand Duchy of Baden, was considered by medical observers as one of the best constructed asylums east of the Rhine, besides being so well conducted as to have merited the approval of various foreign visitors, who had inspected that establishment, I therefore resolved to follow their example, and so judge for myself. Although not strictly within the original scope proposed in these desultory notes on French provincial asylums, I have nevertheless been induced to add a brief notice of the institution now named, to the various sketches already submitted for the perusal of my professional brethren; but how far such a step may accord with previous proceedings, others, not the writer, must determine. This much I would still anticipate, viz.,—that the facts and figures about to be detailed, for the use of those readers who may peruse this narrative, will be received as some apology for thus attempting to occupy further attention.

Influenced by the above motives, and hoping the objects proposed in this extended communication will be deemed in part satisfactory, I therefore proceed at once to remark, in reference to Illnau that, formerly lunatics belonging to the Grand Duchy of Baden were placed under medical treatment, first in the town of Pforzheim—containing about 6000 inhabitants, and situated at the confluence of the rivers Enz, Wurm, and Nagold—until 1826, when they were transferred to Heidelberg. This change was, in many respects, considered an improvement, as the vicinity of an university of celebrity served to dissipate various prejudices previously entertained by the public respecting insanity, whilst it increased the zeal of attendants. However, the place selected being surrounded by buildings, having no adequate space for the inmates, either to take bodily exercise, or of being employed, it was soon found to be most objectionable. Besides these grave disadvantages, seeing it became wholly impossible to prevent frequent communication between the lunatics and residents of several houses, in immediate contact with the asylum; and being also impracticable, owing to the limited accommodation afforded, to classify the patients judiciously, or even to separate both sexes sufficiently, the public authorities resolved to select another and more appropriate site, whereon to construct an entirely new institution; in the interim, about 60 patients being taken back to Pforzheim. In 1836, the Baden government decided upon constructing the present asylum of Illnau, which was commenced immediately; and having been finished in 1842, when lunatic patients were first admitted, the establishment has now continued open about ten years.

The situation chosen is near the small town of Achern, nearly eighteen miles north-east from Strasbourg; and placed almost in the centre of the Grand Duke of Baden's territories. The actual position of the Illnau asylum is certainly fine, if not beautiful; since it has on one side the extensive plain in which the Rhine flows, and on the other, but close behind, is bounded by lofty yet highly picturesque mountains. At a distance, but beyond the fertile Briesgau, the Vosges hills in France are seen; whilst those of the Black forest, sometimes rising precipitously to an elevation of 4000 feet, limit the view in an opposite direction. Altogether, the landscape thus afforded appears most splendid, and may well bear comparison with many scenes often much lauded by travellers, who have visited the Alps or Pyrénées. This German institution is, however, not only remarkable for the magnificent and varied surrounding scenery, but likewise for its salubrity. The soil is dry, sandy, and free from damp, or marshy ground, besides being sufficiently covered with trees; and as the neighbouring fields are productive, many of the articles required for food, by a large population, can be easily obtained and in abundance. The healthy looks of most country people resident in this district of Baden, also furnish conclusive evidence that, the legislature and government acted judiciously, when they resolved to furnish the supplies necessary to build a large public lunatic asylum near Achern. Although neither marshes, lakes, nor any large river are found in the vicinity of Illnau, nevertheless, the establishment is abundantly supplied with water; not only from springs within its precincts, but likewise by a moderately deep, yet rapid rivulet, which meanders in the immediate neighbourhood. The water obtained from this source is of excellent quality, and being besides amply sufficient for every necessary household purpose, it is also collected by a pond, made expressly in one of the fields adjoining the asylum, for the purpose of enabling patients to bathe in the open air, or of learning to swim; analogous to the conveniences reported in a previous page, as peculiar to the Dijon departmental institution for lunatics.

Somewhat similar to the asylum at Stéphansfeld, the Illnau establishment consists of a series of separate buildings, having gardens and airing grounds adjoining, but so entirely disunited as to prevent, if necessary, any communication. The court-yards amount to twenty: ten being on the male side, and ten attached to the female division. Possessing such ample means for proper classification, it therefore becomes an easy proceeding to subdivide the patients into numerous sections, according to their individual maladies, and other distinctive qualifications. Each sex, therefore, comprise ten subdivisions, five of these sections being appropriated for curable, and five for incurable lunatics, whilst agitated patients are always placed in the ground-floor apartments. Generally speaking, every dormitory has from eight to twelve beds, although sometimes only four of these are occupied.

The central building contains a large chapel, in which the Protestant and Catholic services are performed alternately on Sunday, by the respective clergymen of both persuasions; of whom two are attached to the establishment. Underneath this sacred part of the edifice, a spacious ball or concert-room has been arranged, where dancing and musical re-unions of the insane residents are held frequently and periodically. This large apartment, or hall, seemed well adapted for the purposes proposed, although it looked rather sombre, in consequence of the paucity of windows, through which, besides admitting more light and air to the assembled audience, a fine view of the mountains and neighbouring scenery would have been also obtained. Apparently some fête had been very recently celebrated in this locality of mirth and amusement; seeing a huge letter L, intended to represent the reigning grand Duke's cypher, composed of flowers, with festoons of evergreens, hung opposite the music gallery. The idea thus portrayed was pleasing to contemplate; whilst the use to which this saloon-looking apartment was dedicated produced equally agreeable impressions. Nevertheless, I could not avoid thinking simultaneously that, the chapel and concert-room appeared thus too intimately united, since religious services and gay music, or dancing parties, are decidedly different in their object and nature. Neither ought they ever to appear in any way physically conjoined, since each are morally separated, and otherwise in opposition as to their effects upon society, wholly irrespective of much higher considerations.

The asylum's exterior is agreeable; and as no iron bars are seen on any window, the entire structure looks more like an ordinary factory, than a building for the accommodation of lunatics; whilst an open lawn in front, with the residences for officers and domestics on each side of this enclosure, give to the institution a cheerful appearance. Nevertheless, if disposed to be hypercritical, it would be respecting the numerous trees which have been planted, in some places, rather too near the dormitories, and hence to interrupt free ventilation; or perhaps to produce damp, which must prove injurious to the health of inmates. Should such effects follow the cause assigned—and that notion is by no means visionary—such influences could be easily remedied by thinning the adjacent plantations.

From Achern to the principal entrance of the asylum an excellent highway, having a gravel footpath, with rows of trees on each side, has been made for the convenience of visitors; and as this avenue leads through fertile green fields, from whence lofty hills are seen looming in the back ground, travellers cannot feel otherwise than much pleased with the impressions produced, on approaching this lunatic institution. Such, at least, were the sensations affecting my own mind the day I visited Illnau, when the weather was fine, and the sun shone out in splendour. A delightful breeze fanned the luxuriant foliage around, which then cast a deep shade over the road perambulated; whereby, the morning's walk became exceedingly agreeable, whilst various surrounding objects took strong hold of my feelings and senses, however devoid I may be of any poetical imagination. Notwithstanding the magnificent weather and splendid scenery, seen everywhere, with the comfortable-looking people I occasionally encountered, still, being a solitary foreigner about entering a German madhouse, in which all would be entire strangers, there appeared such an unusual interest round present proceedings, as to make me pause and contemplate. Now, I was quite a free agent, in excellent health, and permitted to admire nature in true magnificence, without any molestation; but a few moments afterwards would exhibit afflicted fellow creatures in various respects entirely different, although many outward things might seem nearly the same. Such is, however, the chequered life of man. Consequently, persons ought to feel grateful for whatever advantages they may individually enjoy, when contrasting their particular lot—especially if fortunate—with that of less happy or prosperous members of the great human family.

According to existing regulations at Illnau, curable and incurable lunatics of both sexes, and belonging to all classes of society, are admitted as patients; but parties, not natives of Baden, are only received when there is sufficient room in the dormitories. Idiots, epileptics, and cretins, as also lunatics affected with any loathsome disease are, however, inadmissible; the old asylum at Pforzheim being especially consigned for their reception. Indigenous curable insane patients are uniformly admitted in preference to any other persons; but incurables can be only received as inmates when indigent, and considered dangerous. An important rule in reference to the admission of curable pauper lunatics deserves particular notice, on account of its beneficial operation: namely,—wherever application has been made for admission in such cases before the patient's mental malady has continued six months, then no payment is exacted from the commune for their maintenance during the first half years' residence. The object of this excellent regulation being to induce relatives, or others, to send such insane persons to the asylum without delay, so as to augment the probability of ultimate recovery.

The medical staff attached to the Illnau institution consists of one chief physician, Dr. Roller, who is also director; two physicians, one being for the female, and another for the male division, with two internes; all being resident. Besides the above officers, there are occasionally medical pupils in attendance, who may also reside in the establishment, on paying a fixed sum for board and lodging. This feature in the arrangements at Illnau originates from a recent law made by the Badish legislature, which makes it imperative for every medical practitioner—desirous of obtaining any official appointment under government—to have first attended as a pupil in some lunatic asylum during at least three months, in order to acquire practical knowledge based upon experience, respecting the nature and treatment of mental diseases. This constitutes an admirable and most useful regulation, which ought to be adopted everywhere, and enforced by all licensing medical colleges and corporations throughout the British dominions.*

At the period of my visit to Illnau, the insane residents amounted to 410; of whom 206 were male, and 204 female lunatics. Amongst the entire number, about one-sixth were pensioner patients, paying from 400 to 600 "guilders," that is, 32*l.* to 50*l.* per annum; but where the inmate was a foreigner, the annual payment then varied from 500 to 750 "guilders," or 40*l.* to 60*l.* Although many were classed as incurable lunatics, still about one-third of the total patients appeared curable cases, their mental malady having only recently supervened. Several paralytics were likewise under treatment, although that form of insanity seemed by no means frequent in this asylum. In nearly every part of the establishment, the bedsteads were principally made of wood; iron not having been to any extent yet introduced. The cells for the reception of excited lunatics, when seclusion was considered advisable, seemed well ventilated, and even cheerful-looking apartments, being by no means like the dungeons which were so common during olden times in most countries of Europe. Indeed, these receptacles appeared superior to many seen elsewhere, although they certainly cannot be placed upon a par with the cells recently constructed at either Châlons or Stéphansfeld.

Judging from their outward physical aspect, when perambulating the various divisions of this institution, most of the residents seemed to enjoy good corporeal health; whilst very few inmates were observed then under treatment in

* Since writing the preceding paragraph, I am much pleased to report, through the recommendation of my experienced friend, Dr. Scott—examining physician to the East India Company, the board of directors have decided that, in future every medical officer, nominated for their service, must have attended as a pupil at some public lunatic asylum, in order to study insanity and its treatment, during at least three months, previous to undergoing an examination for such appointments. This new regulation is highly commendable: and I trust the governing authorities at home—naval as well as military—will soon imitate so excellent an example.

the infirmary for any bodily disease. Respecting the application of restraint in excited maniacs—which is always a true indication of the system pursued in treating lunatics—although not so frequently employed as in several French and some German asylums which I could indicate, still the proportion of cases where personal coercion was used seemed greater than the ratio recorded in previous pages, at various public institutions: seeing five female and three male patients were confined by strait-waistcoats on the day of my visit to Illnau. All were otherwise free and unrestrained; but, in extenuation of such practices, it should be remembered that, many foreign physicians sincerely believe there is greater liberty, if not kindness and safety towards the patient, when an excited maniac is restrained by a loose camisole, than if placed in solitary confinement, or even committed to the special care of one or more attendants. This mode of proceeding they consider often proves in a higher degree irritating, than using a strait-waistcoat. At least, such is the conviction of some distinguished continental practitioners.

During the year 1850, the following official return exhibits the movement of insane patients at the Illnau asylum:—

| | Males. | | Females. | | Total. |
|---|---|---|---|---|---|
| Admitted . . . . . . | 86 | ... | 71 | ... | 157 |
| Discharged Cured . . . | 33 | ... | 28 | ... | 61 |
| Died . . . . . . . . . | 16 | ... | 9 | ... | 25 |

From the above statement, it appears that, more male lunatics were admitted into the institution than females; but, although the comparative ratio of recoveries in both sexes were nearly identical—about 40 per cent. compared with the admissions—a much larger number of the former died during last year than amongst the latter class; the relative proportion of deaths being 18·60 per cent. of male, and only 11·26 per cent. of female patients, both being calculated according to the actual amount admitted. Besides the practical importance of these data, the figures now quoted are also instructive in another respect, seeing the number of male lunatics received, confirm the accuracy of the opinions enumerated by various observers respecting the greater frequency of insanity, met with amongst men than women, in many districts of Germany. As further evidence, in proof of these conclusions, it may be also stated, when the insane patients were removed from Heidelberg and Pforzheim, during 1822, to the new asylum at Illnau, they consisted of 181 male, and 133 female inmates. Again, two years afterwards, when the total residents of the latter institution amounted to 382 lunatics: they comprised 208 males, and 174 females, whilst the additional patients admitted during that period, comprised 66 of the former, and 60 of the latter sex; thus giving corresponding results to those more recently recorded. From these facts, it therefore seems established that, mental disease oftener affects the male than the female portion of the population, in this part of Deutschland.

Possessing a small farm of forty acres, in addition to the gardens adjoining, means are thereby supplied for employing patients, to a certain extent, in agricultural and horticultural pursuits, especially, as the out-door occupations are held in considerable repute at this asylum; not only for both sexes belonging to the lower ranks, but likewise for patients in even the more elevated class. Besides different employments in the open air, having various workshops attached to the institution, numerous inmates may be frequently seen busily employed in a variety of trades and handicrafts. Thus, tailors are often observed at work, also shoemakers, carpenters, locksmiths, turners, cartwrights, and bookbinders; in short, every means are adopted to occupy patients, compatible with their physical powers and mental health; seeing such proceedings often prove highly beneficial. The work thus performed is also of great importance to the institution in regard to economy, as, for instance, most of the wearing apparel required by inmates is made upon the premises. Cutting and storing the large quantity of firewood, used in such an extensive establishment, also occupies a number of male patients during the summer season. The female inmates likewise labour with as much zeal as the other sex; many being constantly engaged in ordinary household duties, others are busy at various kinds of needlework, or in plaiting straw, which forms a common and favourite occupation to females in the Rhenish provinces.

But mere physical labour is not the only extra-medical measure employed to aid other means of treatment. Excursions beyond the asylum precincts, besides various kinds of amusement being often called into requisition: whilst music, and different varieties of intellectual occupations are frequently used with advantage, especially to rouse and strengthen the lunatic's dormant faculties. Hence, musical reunions assemble under the direction and tuition of professional instructors, which not only become the source of great gratification to a people like Germans, who are all lovers of sweet sounds, but the effects prove otherwise salutary. With reference to intellectual pursuits, those in most repute at Illnau, seemed to be reading, writing, arithmetic, and geography. Besides the above accessories, frequent promenades take place, which are occasionally, in fine weather, so numerously attended that the establishment has been left almost empty of occupants. These excursions are not always confined to the vicinity of Illnau—however beautiful the surrounding scenery—as they are sometimes extended as far as Kehl, or even to the environs of Strasbourg, eighteen miles distant, which is remarkable for its unrivalled spire—the highest structure in the world—besides extensive, and almost impregnable fortifications. Parties of lunatics, occasionally, also visit the beautiful cascade, near an old convent, denominated "All Saints," which is situated high up in the neighbouring mountains, and from whence may be seen one of the most interesting prospects throughout Rhine-land.

Reviewing the various impressions produced upon my mind when visiting Illnau, I can justly say they were favourable to the establishment; whilst the conclusions then formed have since been strengthened by subsequent reflection, and additional information more recently obtained. The inmates appeared to be sedulously superintended, looked healthy, and in good bodily condition; at the same time that order and tranquillity seemed to reign throughout the institution. To say the court-yards and dormitories were less noisy, especially on the female side, than in several French asylums, might be anticipated almost as a matter of course: considering the marked difference manifested in some essential peculiarities of character, which distinguish the two people. Betwixt the imaginative, ardent, and often volatile but intelligent natives of France, and the phlegmatic, contemplative, laborious, and domestic Germans, there often prevails such decided discrepancies of conduct and disposition, that it is quite natural to expect residents in lunatic asylums will behave, under similar circumstances in both countries, as unlike each other as they generally do in sane society. To Stéphansfeld and its inmates the population of Illnau, as also the various buildings, undoubtedly exhibit considerable resemblance; but this feature becomes less remarkable, when it is remembered the residents of both asylums possess many peculiar features in common; seeing they were originally almost the same people. Compared, however, with the lively and impressionable natives of the Orleanois, of Anjou, or those dwelling farther south, it cannot appear singular if lunatic patients, belonging to Alsace and Baden, should be more sedate, even when confined in a madhouse, than persons afflicted with insanity who were born in warmer regions, besides being endued with very different feelings, temperaments, and physical constitutions.

Contrasted with many similar institutions for the insane in Germany, that of Illnau is far superior, both in respect of accommodation, mode of management, and the moral treatment now pursued. I might allude to several, from personal observation made during former years; but great improvements having been effected since that period in most of these establishments, any comparison, at present, would therefore be neither correct nor equitable. Nevertheless, in reference to the public lunatic asylum of Vienna, which constitutes a separate division in the large "Krankenhaus" of that city, there is less objection to my comparing the two together, seeing the latter establishment was, recently, very much in the same condition it exhibited many years ago, when I visited the Austrian capital. The "Irrenthurm" of Vienna then appeared the very worst receptacle for lunatics I had ever, previously or have since, inspected; many of the inmates being bound by chains, and howling in dens, more like wild animals in cages than christian men: whilst numbers had almost nothing but straw for their covering. Being a circular tower, five stories high, any noise made in the lower part of this building could be easily heard in every upper apartment; so that, however quietly the residents of that portion might behave, it was nearly, if not utterly impossible to remain tranquil, or enjoy repose. Further, as a court-yard for patients taking exercise occupied the centre of this cylinder-like structure, its position hence became, in every way, most objectionable. Again, the floors, as well as the ceilings of the cells, being stone-arched, the whole arrangements were cold-looking, sombre, and truly comfortless. Indeed, I may assert, without exaggeration, nowhere else has such a badly adapted institution for the reception and treatment of lunatics ever come within my personal observation in any part of Europe I have visited: and it is hoped fate will never let me see the like again.

Having been built in 1784, when public asylums for the insane were often much worse than prisons, generally unhealthy, usually very imperfectly ventilated, and always filthy, the fact cannot therefore appear surprising, should the old madhouse of Vienna still exhibit, according to observations published by late travellers, some of the revolting features which were so forcibly portrayed at the period of my visit to that institution. A new asylum having, however, been recently built in this capital, at an expense of £80,000, it constitutes the largest, and one of the best conducted receptacles for insane patients throughout Germany; and, as Dr. Riedel—formerly medical director of the lunatic establishment at Prague—has been appointed the chief officer, that seems a sufficient guarantee this asylum will be so managed, in future, as to place it on a level with many others of the highest repute, whether in France or England. Believing such will be the case, and trusting also, that farther ameliorations will be likewise made at the old "Irrenthurm" of Vienna, I must here conclude this rather brief report respecting Illnau, by remarking, as the latter asylum excels many other insane institutions of Germany, which might be easily named, its superiority consequently confers greater honour on the government and legislature of Baden, by whom this public receptacle for lunatics was established: but especially upon Dr. Roller, who has so materially contributed to place the asylum in the high position it now, deservedly, occupies throughout Europe; to say nothing of the new efforts continually made to accomplish additional improvements.

### GENERAL REMARKS.

Having now brought to a close my rather lengthened report on the respective asylums inspected during last autumn, before considering the facts obtained at the different institutions, in the aggregate, I would make one preliminary remark that several of those which have been only recently constructed were, if compared with others built at an earlier period, of a very superior description. This opinion applies especially to Châlons, Auxerre, Dijon, and Stéphansfeld, as also to Illnau. Considered as a whole, in reference to structure and internal arrangements, Stéphansfeld was, however, superior to all the others, according to my judgment; although the new dormitories at Châlons appeared certainly of a better description, than similar apartments elsewhere. At Auxerre, the female division is excellent; and when the proposed new buildings are completed, that asylum, I feel confident, will then become one of the best in all France, from its superior accommodation. The exterior of Illnau, and the judicious arrangement of its various court-yards deserve much praise; but, internally, it must yield the palm to Stéphansfeld and Auxerre. Again, Dijon and Auxerre possess great advantages, in having an ample supply of water in each court-yard; whilst, at the first-named institution, it is even conducted by pipes to the different dormitories. The important benefits consequent upon always possessing an abundance of water, in every receptacle for lunatics, are so universally acknowledged, that visitors will admit these two asylums should be held up as models for others to imitate, if unable to surpass them both, in respect of that most essential element in populous establishments.

Although the asylums enumerated in the previous paragraph were considered superior to many others in respect of their physical capabilities taken altogether; nevertheless, in one or two points, several of the other lunatic institutions are indubitably excellent, and deserve commendation. For instance, the dormitory and really beautiful garden, for dirty female patients, at Maréville, surpassed anything of the kind observed in other asylums; whilst some of the court-yards at Clermont appeared more open, spacious, and better adapted for their specific purposes than similar enclosures in several other localities. Fains was truly *fair*, and its gardens beautiful; but Armentières certainly could not be put in comparison with any, being much inferior; whilst Lille, notwithstanding the zeal and attention of various officers, seemed totally unfit for the purposes of an asylum: not only on account of many irremediable inherent defects, but from its objectionable situation. These evils have been recently rendered much worse than before, by the new station of the northern railway, whereby incessant disturbance and confusion prevail in the streets, which bound three sides of that asylum. All this the lunatics hear, at the same time that their screams, and the agitation incident to 335 noisy female lunatics, may be frequently recognized by crowds of passengers. Comparisons often appear odious, but justice to all parties must rise superior to such considerations; therefore, when placing Lille at the bottom of any comparative scale, and Stéphansfeld on the pinnacle, it ought to be always remembered, the former was anciently a religious house; whereas the latter is a new asylum, built expressly for the reception of insane patients.

Considering it might likewise prove instructive, and, at the same time, enable inquirers to deduce inferences respecting the several institutions referred to in previous pages, with greater facility, if the various facts now recorded were arranged in such a manner as to present, at one view, a general statement: I have, therefore, compiled the subsequent table. Seeing the returns embrace so large a number of patients as 4604 resident in the different asylums, at the period of my recent visit, and that it likewise gives the total admissions, cures, and deaths actually reported during 1850, I trust the document will be considered interesting, even although some readers may only see therein a mere repetition of figures, with which they were before sufficiently familiar.

*Table shewing the Movement of Patients in Nine French Provincial Asylums, and one German, during* 1850. *Also, the total Insane resident Population, when visited in August or September*, 1851; *with the Number of Persons under restraint.*

| NAME OF ASYLUM. | MOVEMENT OF PATIENTS IN 1850. | | | | | | | | | TOTAL POPULATION IN THE AUTUMN OF 1851. | | | PATIENTS UNDER RESTRAINT. | | |
|---|---|---|---|---|---|---|---|---|---|---|---|---|---|---|---|
| | ADMITTED. | | | CURED. | | | DIED. | | | | | | | | |
| | M. | F. | Total. | M. | F. | Total. | M. | F. | Total. | M. | F. | Total. | M. | F. | Total. |
| Armentières . . . . . | 123 | — | 123 | 29 | — | 29 | 58 | — | 58 | 496 | — | 496 | 15 | — | 15 |
| Lille . . . . . . . . | — | 84 | 84 | — | 16 | 16 | — | 25 | 25 | — | 335 | 335 | — | 28 | 28 |
| Clermont . . . . . . . | 132 | 134 | 266 | 20 | 33 | 53 | 68 | 85 | 153 | 390 | 486 | 876 | 4 | 14 | 18 |
| Châlons . . . . . . . | 37 | 36 | 73 | 30 | 15 | 45 | 13 | 8 | 21 | 145 | 164 | 309 | 4 | — | |
| Fains . . . . . . . . | 36 | 24 | 60 | 15 | 9 | 24 | 22 | 8 | 30 | 186 | 155 | 341 | 1 | 3 | 4 |
| Auxerre . . . . . . . | 36 | 39 | 75 | 11 | 11 | 22 | 15 | 10 | 25 | 109 | 157 | 266 | — | — | — |
| Dijon . . . . . . . . | 42 | 50 | 92 | 15 | 13 | 28 | 6 | 14 | 20 | 101 | 153 | 254 | — | 5 | 5 |
| Maréville . . . . . . . | 108 | 90 | 198 | 24 | 17 | 41 | 44 | 40 | 84 | 471 | 405 | 876 | — | 3 | 3 |
| Stéphansfeld . . . . . | 82 | 73 | 155 | 17 | 18 | 35 | 17 | 15 | 32 | 220 | 221 | 441 | 1 | — | 1 |
| Illnau . . . . . . . . | 86 | 71 | 157 | 33 | 28 | 61 | 16 | 9 | 25 | 206 | 204 | 410 | 5 | 3 | 8 |
| TOTALS . . . | 682 | 601 | 1283 | 194 | 160 | 354 | 259 | 214 | 473 | 2324 | 2280 | 4604 | 30 | 56 | 86 |

According to these statistics, speaking generally, a larger number of male patients were admitted than female; the excess being 81, or 13·47 per cent. of the former over the latter sex. More males than females were also cured; the ratio being 28·44 per cent. of that class against 26·62 per cent. of the opposite. Again, the proportion of deaths predominated amongst male lunatics, of whom 37·97 per hundred admissions died; whilst the mortality amongst female inmates amounted to 35·60 per cent, similarly calculated. Respecting the total population, at the period of my inspection, although considerable discrepancies prevailed in particular establishments, male patients on the whole predominated; the excess being 44, or less than 2 per cent. In reference to the aggregate number of inmates under treatment, it may be noted as a rather curious coincidence, that the two largest asylums named in the table contained identically the same amount of patients, although the ratio of the two sexes was different. Thus, Clermont and Maréville have each 876 lunatics, the majority in the former asylum being female patients; whereas, at the latter institution, male inmates were most numerous. On the other hand, in the great valley of the Rhine, which comprises Alsace and Baden, it certainly appears very remarkable that, in the only two public asylums for the insane of these provinces, which are, in many respects—both physical and moral—very similar, the sexes of patients then under treatment should be all but equal; seeing, at Stéphansfeld and Illnau 425 lunatic females were then resident, whilst the number of males similarly afflicted was 426 at both places. It is also worthy of notice that, the total admissions were nearly the same in amount at these two asylums, whether compared in reference to sex or number; since the new patients admitted into the former establishment were reported to be 155 against 157 received at Illnau during the parallel year. The gross mortality, however, varied considerably in the different institutions, calculated according to the number actually admitted. Thus the ratio of deaths ranged so high at Clermont as 57·51 per hundred admissions; whereas, at Maréville, where the residents were exactly the same in number, it amounted to 42·42 per cent. At Armentières, into which only male lunatics are received, the mortality was 47·96 per cent.; whereas, amongst the male patients at Dijon, the proportion was less than one-third that amount, or 14·28 per hundred cases admitted. Further, at Châlons, the deaths amounted to 22·76 per cent.; whilst at Auxerre the ratio was 33·33, or one-half more than the latter proportion, speaking comparatively; consequently, the highest mortality recorded took place at Clermont, the lowest being observed at Dijon; in which asylum, both sexes included, the ratio of deaths was 21·73 per hundred admissions.

Respecting the all-important question of personal restraint, a single glance at the previous table speaks more eloquently, and to the point, than any lengthened dissertation. In that document, Auxerre stands pre-eminent, no case whatever being in camisole. Stéphansfeld, in which only one patient was partially restrained amongst 441 lunatics, comes next, being almost on a par with the former institution; whilst Lille occupies the lowest position in this comparative scale, seeing 1 patient, in every 12½ inmates under treatment was confined by a strait-waistcoat. English physicians cannot approve of similar proceedings; but then it should be always remembered, the feelings, constitutions, and habits of the French and English people are different in many particulars. In this country, the temperament of most persons appears less excitable, and more sedate; they are usually very obedient to law and order, although real freemen; besides being oftener submissive to the control of public opinion, and under the guidance of leaders in whose judgment, honesty, and experience they repose confidence. Such are the natural characteristics of most Englishmen when in health, and which seem even to influence their ordinary conduct although insane; whereby they become more readily obedient to superior authority. Beyond the opposite shores of "La Manche," matters have generally a very different aspect. Endowed by nature with much more excitable temperaments and vivid imaginations, being less willing to obey law, excepting through the strong arm of power, having more confidence in their own individual superiority, than willingness to place themselves under the guidance of other men, or submit to external control, the natives of France do, when labouring under mental derangement, frequently show that such motive springs of action still predominate. To my mind, at least, it has hence appeared as if sufficient importance was not always assigned, by foreign observers, to the difficulties now described, but against which French medical practitioners have very frequently to contend, during the treatment of excited maniacs. Consequently, when recording the greater application of personal mechanical coercion in that country, critics ought never to forget the above manifest discrepance of character, which distinguish the respective nations. On that account, greater credit is really due to MM. Girard, Dagonet, and Morel, for the very successful efforts they have recently made in promoting the non-restraint system. Like the learned Baglivi, when alluding to the diseases of Rome, a Frenchman may justly say, in reference to the camisole, which is still too often applied by his countrymen, 'Scribo in aere romano.'"

The greater liability of one sex to insanity more than the other, has lately occupied consi erable attention, both in this country and on the continent. From official data recorded in a previous page, it has been shown that, throughout several districts of France male lunatics were most numerous. Since geographical position would seem to exert considerable influence respecting this question, I have been induced to construct the subjoined table, which includes four lunatic asylums in the northern departments, so as to contrast the results thus recorded, with four similar institutions belonging to the central provinces, although some were, however, visited during my previous excursion, published in a former volume of the "Psychological Journal."

*Table illustrating the liability of the two sexes to Insanity in Northern and Central France.*

| Northern France. | | | | Central France. | | | |
|---|---|---|---|---|---|---|---|
| Asylum. | M. | F. | Total. | Asylum. | M. | F. | Total. |
| Armentières . . | 496 | — | 496 | Nantes . . . . | 181 | 210 | 391 |
| Lille . . . . | — | 335 | 335 | St. Gemmes . . | 161 | 179 | 340 |
| Fains . . . . | 186 | 155 | 341 | Orleans . . . . | 246 | 275 | 521 |
| Maréville . . . | 471 | 405 | 876 | Dijon . . . . | 101 | 153 | 254 |
| | 1153* | 895 | 2049 | | 689 | 817† | 1506 |

From the above statements, there cannot remain any doubts respecting the greater tendency of females to be attacked by mental disease, in the central than northern departments; where an opposite result generally obtains. The larger number of insane females under treatment, compared with male lunatics, at the public establishments of Charenton, Bicêtre, and the Salpêtrière, also indicates the same peculiarity prevails in Paris. Thus, on the 1st of last January, the total number of male lunatics in the two former asylums, and at the succursal farm of St. Anne, were 1,082; contradistinguished to 1543 insane females under treatment, at the same date, in Charenton and the Salpêtrière; thereby showing an excess of 461 patients amongst the latter sex, or 42·60 per

* Giving an excess of 258 males, or 28·22 per cent.
† Giving an excess of 128 females, or 16·04 per cent.

cent throughout the department of the Seine. Such result being nearly similar to the observation recently made in the British metropolis, where, female lunatics likewise predominate considerably.

Political and religious excitement, or important questions which intensely occupy public attention often produce, it is well known, marked impressions upon the minds of large classes of people; consequently, it cannot seem surprising, should the susceptible organizations of certain individuals suffer during popular commotions. At the period of the crusades and great Reformation, during the first French republic, or subsequent wars in Italy and on the Rhine, the truth of this observation was unequivocally demonstrated; especially in reference to the production of insanity. When the Emperor Napoleon upset dynasties, and overran Europe, imaginary kings and princes were numerous in the asylums of Germany and France, of which various examples have been recorded by authors; amongst whom may be cited Pinel, who states that, three Louis the Sixteenth maniacs were at the same period under treatment at Bicêtre. Again, during the recent revolution in France, similar results have been observed in reference to the origin of mental disease; and I may mention that, in several asylums, imaginary Préfets, self-styled representatives, and other fictitious high personages, who had lost their senses during the late political disturbances, were met with amongst the inmates. At one institution described in previous pages, there recently existed three maniacs who believed themselves to be Louis Napoleons, and consequently all presidents of the Republic. In another institution, one ideal Louis Napoleon, was also under treatment; whilst candidates for the presidential chair, or seats in the legislature, and others who believed they occupied official appointments—each poor creature being insane upon a particular subject—were occupants of departmental asylums. These facts are instructive, and shew, wherever the population of a country become excited by exalted predominant ideas, especially amongst persons predisposed to insanity, and otherwise of weak nervous organization, they will, most likely, suffer from the influence of such causes, which have been occasionally considered by some philosophical observers, as a mental epidemic.

Although it was not originally proposed in the present remarks to discuss the medical treatment usually pursued at French asylums, one point seems, however, of so much importance that, it deserves some notice in these pages; particularly, as great unanimity of opinion prevails amongst the physicians of departmental institutions, with whom I had an opportunity of conversing upon the question. I now refer to employing blood-letting as a remedy, in cases of insanity. Without an exception, every practitioner was decidedly opposed to the general abstraction of blood in maniacal patients; as they considered it not only unnecessary, but often highly injurious. In many cases, venesection produced so much subsequent depression, that attacks of mania, which otherwise might have been of short duration, under a different, but more judicious mode of treatment, were thereby prolonged, and even ended in fatuity. Numerous examples were pointed out, during my recent and former visits, of insane patients being bled previous to their admission into asylums, but who, instead of losing blood, ought rather to have been better nourished, in order to restore their physical strength, besides having tonic remedies prescribed to counteract the existing nervous debility, which produced their delirium, and consequent excitement. Of course, particular instances of insanity presented themselves where inflammatory symptoms appeared so decided, or in which apoplectic congestion existed to such an extent that, local or general abstraction of blood was then absolutely necessary; nevertheless, these examples were exceptional, and only confirmed still further the observations made by the most experienced medical officers of French asylums, respecting the baneful consequences of blood-letting, in most cases of mental disease, which came under their cognisance. Indeed, one gentleman remarked, "the delirium of insane patients was never modified by frequent and copious bleedings, but often the reverse."

Being supported in these practical conclusions, by the opinions of many English physicians, it cannot be too strongly impressed upon the minds of young practitioners, or of those who may not have had much experience in treating cases of insanity, to be always exceedingly chary of using the lancet, as blood once abstracted, cannot be speedily replaced; while the depression thus produced upon the system is not temporary, but often very permanent, and hence highly detrimental. Where blood-letting is thought necessary, tartar emetic will frequently prove in a higher degree advantageous; seeing the debility thereby produced, and its peculiar action upon the patient's frame soon cease, whenever the remedy is discontinued. This preparation of antimony is also very useful in both apparently and really inflammatory cases of mania, affecting strong muscular or plethoric patients; and I firmly believe, if tartar emetic was oftener used, instead of abstracting blood, the results would be much more satisfactory.

### ADMINISTRATION OF DEPARTMENTAL ASYLUMS IN FRANCE.

Considering it will be esteemed interesting to English readers of these notes, if some details respecting the executive, and mode of administering public lunatic institutions in France, as now generally pursued, were added to previous observations, I have been induced to make inquiries on such subjects; in order to point out several evident defects in the present system, although at the same time various matters deserve marked commendation. Should my subsequent observations be ever noticed by French authorities, they must be taken merely as the independent, but well-meant criticisms of an Englishman anxious to correct proceedings which he thought defective; especially, in reference to the position of professional gentlemen attached to these institutions, who, speaking from frequent personal interviews, are truly a most meritorious class of officers, and through whose continued exertions, many of the public lunatic asylums of France have chiefly attained the prominent position they now occupy in Europe. Nay, I sincerely think, were these devoted public servants more liberally remunerated, possessed greater executive power, and were less trammeled by local functionaries—sometimes wholly ignorant of insanity—the afflicted patients committed to their medical surveillance would be materially benefited.

Without including such institutions as Clermont, which is private property, or Bon Sauveur, at Caen—belonging to a religious body—whereby large sums are realised from lunatic persons placed in similar establishments, there are at present forty departmental asylums throughout France, appropriated for the reception and treatment of insane patients. Some of these public receptacles have been constructed since the new laws respecting lunacy were passed in June, 1838; although a considerable number were formerly civil hospitals, mendicity depôts, ancient convents, and even military barracks, which had been, more or less appropriately, altered for receiving lunatics. Upwards of half the existing asylums are situated in or quite close to the capital of its own department; others in the chief town of an arrondissement; some even in a cantonal village; whilst a very few are located in rural districts. These institutions are departmental property, having been purchased or constructed at the public expense: and their annual revenue consists almost solely of the payments received from different communes, or other parties, for the maintenance of and treatment of lunatics there resident: which allowance, in the case of indigent patients, amounts, upon an average, to one franc per diem. Private patients, however, pay much higher sums, as already frequently stated in the previous narrative.

The lay administration of public asylums consists of one resident director, who receives a fixed annual salary. He is assisted by the committee of surveillance, which comprise five members; the latter, however, give their services gratuitously. In twenty institutions amongst the forty now enumerated, the chief physician also fills the office of director; and this arrangement is considered judicious, whenever the total patients do not exceed 350 or 400 inmates. At many establishments there is a receiver and steward; but in some instances both these offices are united, similar to the physician-directors. Such an union seems, however, highly objectionable, and it is even said, has been occasionally productive of serious abuses. The Minister of the Interior appoints every director, physician, receiver, and steward, besides the committee of management; and all internes, excepting in cases where the budget of expenses does not exceed 100,000 francs: under which circumstances, the préfet of the department nominates. The almoner is always elected by the bishop of the diocese, and he must reside in the asylum. In a number of institutions religious sisters, assisted by laical servants, superintend the laundry, kitchen, dormitories, and even the pharmaceutical department; although there are various asylums without any sisters of charity, all the domestics being then of the ordinary description.

According to these statements—obtained from an authentic source—it therefore appears, that the personal staff of a lunatic institution varies considerably, and hence it would prove very difficult to introduce everywhere any uniform system, which has, it is said, occupied the serious attention of government during the last twelve years; notwithstanding this fact, parties are disposed to believe, even if uniformity were more generally introduced, the plan would not long continue.

With such an organization as now described, besides the heterogeneous elements often composing local authorities, conflicts of interests or opinions frequently become inevitable, and have therefore unfortunately occurred at several establishments. Even instances might be cited where, from the first opening of the asylum, now eight or ten years, a kind of domestic warfare has constantly prevailed. Resignations, changes, and even dismissals, have supervened in asylums disturbed by such feuds; so that, nothing is often so uncertain, as the position of a medical officer. Seeing the modern treatment of insanity does not consist solely in the administration of medicines: but to prove efficacious, constant attention must be also given to patients during the hours of work, at meals, and in their recreations—nay, even during sleep,—these important questions a non-medical superior officer cannot comprehend. Consequently, in order to prevent misunderstandings, and likewise that the executive of large lunatic establishments, may act harmoniously: it has become the practice of recent years to appoint medical men to the office of director, who are hence able to understand, besides their own administrative duties, questions of hygiene, and those ameliorations which may be proposed by the attending physicians. This plan has been advantageously adopted at Maréville, where Dr. Renaudin is now director, having been formerly the chief physician of another asylum: and also at Saint-Yon, near Rouen, in the person of Dr. De Boutteville, who was recently attending physician of an asylum, but is now director of that extensive establishment. In both instances the alteration thus effected has proved highly beneficial.

Although, in many respects, useful officials at lunatic asylums, the religious sisters, and even the almoners, are occasionally carried away by too great zeal, which leads them injudiciously to interfere with the physicians' proper professional duties. In some cases, images, pious books with engravings, beads, scapularies, and so forth, are improperly given to patients, without the medical attendant's knowledge, whereby injurious excitement may be produced, especially when the lunatic is affected with religious delirium. Should the physician forbid such proceedings, the cry of impiety is raised, and even quarrels ensue. Sometimes also the effects of religious exercises are exaggerated, and improper interference made to promote their continuance, which proves equally hurtful.

Being members of a body who have interests and inclinations beyond the asylum, and are often actuated by a desire to support the privileges of their order, some religious sisters hence become like persons serving two masters—the one worldly, the other of a more sacred character. This feeling tends to inconvenience, and may induce such parties to endeavour to counteract the chief authority, of which the following example will supply an apt illustration. Some years ago a new director was appointed to an important asylum. When on his way to take possession of office, he first paid a visit to an old friend residing at a neighbouring town, where he happened to meet the superior sister of charity attached to the establishment in question. The host having purposely avoided introducing the new director and superior to each other by any official designations; they consequently were ignorant of their respective positions; at the same time, however, he led the conversation, so that the asylum and its management soon came under discussion. Amongst other remarks, the lady naively said, "*Apropos*, a new director is expected; but we shall continue to keep the upper hand, as they say he is a mere man of straw." Subsequently, when they became better acquainted, this speaker was undeceived, as the director—an energetic officer—soon got rid of her manœuvres, as also of others similarly disposed, although the disagreement thus created only terminated by his accepting another appointment. This anecdote—quoted on good authority—shows how the system sometimes works injuriously. However, the worthy sisters are frequently meritorious assistants in public establishments, and often do much good; nevertheless, it seems undesirable they should be connected with any power acting externally, or, as it were, behind the throne, that being detrimental to true discipline, essential in every lunatic institution. To my mind, all the sisters of charity, or upper attendants, should be like those at the Auxerre asylum, where they do not belong to any religious corporation out of doors, but are entirely amenable to the resident authorities. This had the happiest results, since matters proceeded much more amicably; whilst there was no restraint at that institution.

Disagreements also arise betwixt the executive and the committee of surveillance, owing to the dislike provincial powers generally entertain against centralization. For example, the director or physician appointed by the Minister is often a young man without much status or fortune, and frequently a perfect stranger to the place, or even altogether unknown in the department. He arrives, and takes possession of a post where he has often no friends, or persons likely to give him support, in the performance of often arduous duties. Being thus circumstanced, and perhaps occupying a situation which was much coveted by the protégée or relative of some person having considerable local influence, he receives the reverse of a warm reception; and instances are even upon record, where councils-general have printed in the official report of their proceedings that, they accepted the appointment of M. X—— as ——, only in consequence of his having been imposed upon the department by central authority. To indicate the annoyance which the superior officers of asylums sometimes endure from such refractory powers, I may mention that the very day of my arrival at Nancy, previous to visiting Maréville, the council-general of the Meurthe—then in deliberation—refused to allow 600 francs for the salary of a third interne, recently appointed by the Minister, although his services were absolutely required in an asylum having 876 patients. However, the young official cannot ultimately lose his allowance, seeing he held the appointment under government, and would receive the amount due by an order of the Minister, which must be obeyed!

The committee of management is usually composed of rich proprietors in the neighbourhood, of members belonging to the council-general, judges of the Court of Appeal, attorney-generals, and other high functionaries, who frequently wish to govern everything, or to act independently of the directing authority. The receiver-steward is almost always a native of the department, and he generally looks to the committee of surveillance as sole superior, or masters; who in turn favour his pretensions, so that he becomes a very independent, if not often the most influential personage in the asylum. Hence, he is apt to interfere in the director's department, or even with the resident physician. Such results are not uncommon; and in ten establishments which could be enumerated, it is stated, eight have become the arena of similar unpleasant dissensions. When these disputes attain to any height, the Préfet is sure to receive complaints, or confidential communications, and then voluminous reports follow. In short, the household being divided, the Préfet becomes mystified as to who is in the wrong, which proves always detrimental to the director or physician's position and authority. Sometimes he sternly turns a deaf ear to these intrigues, or honestly seeks to know the truth. Being, however, often surrounded, or earwigged by influential parties in the locality, who anxiously wish to get rid of the foreign official, he becomes constrained to interfere, and at last applies to the Minister to remove the obnoxious functionary. Occasionally, the persecuted party ends the matter by resigning, and retires to private practice. In other cases it has occurred that the Minister, justly annoyed by such references, decidedly supports his nominee against the manœuvres of provincial schemers, and will neither remove the officer complained against, nor separate the functions of physician and director when united; which frequently forms a bone of contention amongst conflicting powers.

As examples are better than assertions, I will relate two illustrations which actually occurred. In a certain asylum, whose name it is unnecessary to mention, the committee of surveillance, the receiver-steward, and sisters of charity, having obliged the physician-director to resign, the council general of the department, amongst whom there happened to be one or two members of the managing committee, petitioned the minister to separate the duties of director from those of the physician, saying this alteration would end all disputes, and be otherwise useful to the establishment. The minister having yielded, he nominated a director from a distant part of France, and a new physician also, from another locality. Nevertheless, peace was not obtained, as discussions immediately arose with both functionaries. The committee determined not to be out-generalled, prepared a code of regulations, without consulting the new physician, which was afterwards submitted for the minister's approbation; but this was refused. A member of the council, who also had a seat in the legislative assembly, then undertook to arrange matters, and accordingly set off to Paris for that purpose. However, on arriving at the bureau of the Interior, he learned that the committee of surveillance of the asylum was dissolved, and that the Préfet had been ordered by the minister to present a fresh list of members to government for approval. At another asylum, where already two director-physicians had been obliged to yield to powerful local coteries, the same parties even attempted to dislodge a third occupant of the joint appointments, by demanding a separation of offices. The Préfet being constrained, brought the question before the departmental council general, in order to make an impression upon the minister, and so induce him more readily to comply. The matter came under discussion; but although a member stated at this meeting that, the ministerial circular opposed any division of these two offices, unless the patients exceeded 300 in number, and notwithstanding there was no residence for another officer, which the ordonnances required, the vote for a separation was nevertheless carried, and afterwards transmitted to head-quarters. It proved perfectly nugatory, as the minister paid no attention to their recommendation.

Centralization, however productive of various benefits, still, in the opinion of some intelligent medical men, often wants unity of march, and sometimes energy in its measures; consequently, such parties believe that numerous asylums in France are not so perfect as they might be made, were official intrigues less frequent and influential. A friend who knows the subject well, and takes great interest in the management of asylums, when alluding to the defects of several institutions, thus remarked, "At one place the patients are well fed, but badly clothed; while, in another asylum, they have no variety or amusement. Musical entertainments, promenades, gymnastics, and literary occupations, are too often deficient. In some institutions the occupations of patients are not sufficiently organized; and often the patients who work are not properly encouraged, or receive any gratification for the labour performed. As a consequence of this mode of proceeding, no savings become realized, and the inmates suffer in various ways; whilst the under servants, being often interested in the disputes amongst their superiors, disorder and insubordination supervene." To remedy existing defects, some parties have suggested considerable modification, if not the total abolition of councils of surveillance, seeing many institutions have no fixed property to administer; besides which, as the Préfet, sub-préfets, attorney-generals, mayors, presidents of the court of appeal, and of inferior tribunals, as also both inspectors-general, have their eyes constantly upon departmental asylums, the committee in many cases becomes superfluous. Where such a body is considered necessary, it should always consist of practical members accustomed to business. Some should be certainly medical men, although at present—and singular enough—they are nearly always excluded. Architects, merchants, and persons in trade like the former, are also rarely appointed.

Without disparaging present functionaries, none are better qualified for investigating hygienic improvements, renovating old, and deciding upon new constructions, or for inspecting the stores supplied, and seeing the provisions consumed were of good quality, without being overcharged, than the individuals just named, who would be far better superintendents than parties otherwise constituted. In the opinion of another intelligent friend, many of the existing evils would be in a great part remedied, were the official allowances and salary more under ministerial control, besides being at the same time susceptible of advancement. The authority now quoted further says, "The directors, physicians, stewards, and receivers, according to their respective merits, and the duration of service, should pass from inferior establishments to those of higher importance, with increased remuneration. This plan would put an end to various intrigues, and prevent locally connected stewards or receivers from obtaining too much influence, to the detriment of directors and physicians; who, from being often strangers to the locality, are consequently sacrificed. Such changes in the system pursued are indispensable, in order to elevate and improve the administration at present pursued in various asylums; whereby, some have hitherto failed in regard to regularity, dignity, and philanthropy." Similar sentiments are also entertained by other individuals; and, conceiving it might prove advantageous, I have given their opinions a place in these notes; trusting the remarks now made may induce those who have official power, to correct whatever is still defective in a system which certainly possesses many advantages, and has already effected much good to the lunatic population, throughout most parts of France.

As some readers may not be altogether cognizant of the formalities required, when it becomes necessary to place lunatics in an asylum, before concluding my observations on these establishments, I would remark that, the certificate of only one medical practitioner is demanded. Which, however, must be accompanied, if for a private person, by a petition from a near relative, authorizing the party's reception; but, if otherwise situated, an order from the Préfet must be procured prior to the patient's admission. Within twenty-four hours after the lunatic's arrival at an asylum, the resident physician must make a report to the préfecture, containing the chief symptoms of the patient's disease, which he is required to repeat in a more minute manner fourteen days afterwards, as likewise every six months during the inmate's residence. When an insane pensioner is placed by a relative, he may be removed at any time by the same party; but where the case was admitted "d'office," as it is called, the Préfet's permission is then necessary, previous to removal, even where the disease has been cured; but in every instance, whether a private patient or otherwise, if considered dangerous by the physician, none can leave the institution, without the Préfet's express order to that effect. By these regulations, whilst the lunatic is properly taken care of, and cannot be confined longer than the nature of the case warrants, society is also protected against demented persons, who might cause injury to others or themselves.

In consequence of large sums being annually received from private patients under treatment in several departmental asylums, these items often form a very important portion of the ordinary revenue. For instance, at Auxerre, 43,550 francs were obtained during 1850, from this source; and at Maréville, the amount paid by the pensioners, in that year, was 56,837 francs. The profit derived, in this manner, is applied towards improving the respective institutions; and at Maréville, many of the recent alterations have been defrayed by the savings thereby effected. At present, most of the public insane establishments of France are both institutions for indigent lunatics, and "maisons de santé" for members of the upper classes. This arrangement prevails, no doubt, in several asylums of England, but especially in those of Scotland, and proves often advantageous to all parties; although, amongst our neighbours, the medical officers are much more illiberally treated in regard to remuneration received, for the onerous duties thus imposed upon them by the authorities, in attending ladies and gentlemen, at the same time with pauper patients. If the great reputation of Dr. Girard or Dr. Morel, brings many private patients to the institutions of Auxerre or Maréville; whilst the experience of Dr. Bouchet induces rich persons to place their insane relatives under his care, at the Nantes public asylum, the government ought, on that account, in common justice, to grant additional allowances; especially, as throughout every departmental lunatic establishment of France, the medical officers receive inadequate salaries, even where they are, at the same time both director and physician. Taking into account the very responsible position in which physicians of that description are placed, the arduous labour they have to perform, and the large revenues often obtained through their professional skill and knowledge, whilst each are debarred from all private practice, it is mistaken economy, if not great injustice, to pay many of these eminent gentlemen with only three or four thousand francs per annum. Such parsimony is wrong. And should these remarks ever come under the notice of French authorities—whether departmental or belonging to government—as an impartial foreigner, I would strongly impress upon their special attention, the propriety of making some change in this respect; but particularly to take into account the prolonged services of various medical officers in departmental asylums, by whose exertions several institutions have attained the deservedly high position they at present occupy, and to whom much of their actual prosperity may be justly ascribed.

Amongst numerous features usually noticed at the establishments under discussion, and which merit decided approval, none is more beneficial than the appointment of internes to reside in such asylums.

Besides being an excellent practical school—where future practitioners can obtain most important knowledge and experience respecting mental diseases—these junior medical officers become in many ways useful to the physicians, and beneficial to the patients. Having alluded especially to this subject in my former notes, it appears almost unnecessary again to investigate the question, further than briefly to remark that, no public establishment for the insane ought ever to be without resident medical pupils, more or less numerous, according to circumstances. In every case, where internes were attached to French asylums, invariably the duties then seemed performed with greater regularity, and the patients received better attention; whilst the case-books—to say nothing of minor details—

were more accurately kept than otherwise. Upon no point was the utility of internes so remarkably shewn, as in reference to the diminution of personal restraint, amongst the inmates of particular institutions. Wherever an asylum had no resident pupils, as part of its medical staff, camisoles were much more frequently in requisition. Take as examples, Bon Sauveur, having eighteen patients so confined; or St. Gemmes, which had twenty-seven residents in strait-waistcoats, and Orleans, where twenty-five inmates were also personally restrained. To none of these asylums—described in my former notes—were any internes appointed. Again, amongst the institutions alluded to in previous pages, in which these useful pupil-officers formed no part of the establishment, mechanical coercion was uniformly much more frequent, than at asylums otherwise constituted. Thus, at Armentières, fifteen patients were in confinement; and at Lille—also without internes, I saw twenty-eight inmates bound by camisoles. Whereas, at Auxerre, no individual whatever had a strait-waistcoat, and only one patient was so treated at Stéphansfeld; both the latter institutions having, it should be always remembered, intelligent internes. I am therefore so thoroughly convinced respecting the great importance of similar assistants in every lunatic asylum that, none ought ever to be without one or more of these officials. As well might general hospitals be deprived of house-surgeons, clinical clerks, or dressers. All being, as every person knows, not only most advantageous appointments to the young men so employed, but likewise producing great benefit to the patients generally. Uniform observation, throughout France, shews the marked utility of internes; hence, every English asylum should adopt a system which comes recommended by the best of arguments—well-established experience.

### CONCLUSION.

Previous to drawing these general remarks regarding French asylums for the insane to a close, I would observe that, since my recent inspection—described in the preceding pages—an important alteration, in reference to the appointment of physicians to these establishments has very lately been made by the present government, which merits, at least, a passing notice; since it has already created considerable sensation amongst the profession, "d'outre mer," and may eventually produce important consequences, in regard to the treatment of lunatics consigned to departmental institutions. Instead of being nominated, as heretofore by the Minister of the Interior, all medical officers of insane asylums will be appointed in future by the Préfet, who is also empowered to settle their salary and allowances. Under the former régime, those gentlemen who had specially studied mental diseases at Paris or elsewhere, were almost invariably selected by the central authorities, devoid of any local interest; whilst the zeal or ability they manifested, in the discharge of their professional duties, often insured an advancement to more lucrative or important situations, as the reward of continued services. According to the new regulations, it is feared, on the contrary, if physicians of asylums are not only appointed, but placed solely under the Préfet's control, their administrative functions and professional influence will be materially curtailed by these local dignitaries, which may become detrimental to patients, besides diminishing the medical officer's prospect of ever being translated to another establishment, and so obtaining a larger salary with additional attributions.

Writers of eminence in France, besides anticipating the consequences now shadowed forth, likewise think the new decree of the 25th of March, 1852, by which every new medical officer of asylums is made the absolute nominee of "Monsieur le préfet," will, amongst other evils, resuscitate the almost forgotten feuds, formerly too prevalent betwixt the medical attendants of lunatic establishments and religious corporations. An able practical authority, in every question respecting insanity and its treatment—Dr. De Boismont—says, when discussing this subject in a recent publication, "Tout partisan que nous sommes de leur adjonction aux asiles d'aliénés (meaning priests and sisters of charity) nous avouons que, quand les attributions ne sont pas suffisamment délimitées, les corps religieux ont une tendance à s'emparer de la direction de l'administration. Cette remarque a d'autant plus de force, que plusieurs aumôniers n'ont pas hésité á dire que, le traitement des aliénés devait être exclusivement religieux et moral. Cette opinion est une véritable hérésie." Trusting the gloomy anticipations which some have prognosticated, respecting the future administration of departmental institutions for the insane, may prove erroneous; it must be acknowledged, although centralization in many other matters paralyzes individual exertion, and often becomes injurious; nevertheless, it has conferred incalculable benefits upon the lunatic population of France; and that every year, since the law of 1838 was promulgated, important ameliorations have been introduced into provincial establishments, through the medical officers, aided by central power; whilst ancient prejudices are now much more rare; and thousands of unfortunate lunatics have received, as it were, a new life, often rendered more comfortable from improved treatment combined with humane protection. The proposed changes will likely become very disastrous, should the independent authority of the inspectors-general be at all infringed, either in regard to visiting provincial asylums, or in sanctioning and suggesting farther improvements; more especially, since it has been mainly owing to the supervision and controlling influence of these high official gentlemen that, unity of action is now happily established throughout numerous institutions, the zeal of local functionaries excited, merit encouraged and ultimately rewarded, by promoting the most efficient medical officers to other appointments; where they receive better remuneration, besides being placed in a higher public position.

Although not strictly appertaining to lunatic asylums, still, another innovation, which materially affects the profession, deserves being also mentioned; since it shows other organic changes have been made by parties now in power, regarding various responsible offices occupied by physicians. I here allude to the great alteration very recently decreed in reference to supplying future vacancies, amongst professors at universities and colleges, whether medical or otherwise. Henceforth, instead of obtaining those appointments by "concours" amongst competitors, when any chair becomes void, the Minister of public instruction proposes a doctor of medicine who is thirty years of age to the Prince president for election, if the vacant office belongs to the medical department. The minister may, however, select another person from a double list of candidates which is demanded from the faculty where the vacancy occurs; but this proceeding being a mere formality, the choice rests entirely with government. Farther, as Louis Napoleon at present constitutes the chief executive, and of course commands ministers to yield obedience, that autocrat or his satellites will consequently engross the whole patronage. The effect of such regulations can be easily foretold; while they must, doubtless, very seriously influence professional independence, which ought ever to characterise men of science and education.

